# Goal setting with young people for anxiety and depression: What works for whom in therapeutic relationships? A literature review and insight analysis

**DOI:** 10.1186/s40359-022-00879-5

**Published:** 2022-07-13

**Authors:** Jenna Jacob, Milos Stankovic, Inga Spuerck, Farhad Shokraneh

**Affiliations:** 1 Child Outcomes Research Consortium, Anna Freud Centre, 4-8 Rodney Street, London, N1 9JH UK; 2 Euro Youth Mental Health, The Block, 35 Churchgate, Hitchin, SG5 1DN UK; 3 Systematic Review Consultants, 9 Sandfield Road, Nottingham, NG7 1QR UK

**Keywords:** Youth mental health, Anxiety, Depression, Goal setting, Therapeutic alliance, Outcome measurement, Active ingredients, Goal based outcomes

## Abstract

**Background:**

Goal setting and goal-focused work is widely used in young people’s mental health settings. However, little is known about how, why or for whom this is helpful. This study aims to explore the mechanisms of collaborative goal setting as part of therapeutic relationships: is it helpful for young people experiencing anxiety and/or depression, how and why/not, for whom, and under what circumstances?

**Methods:**

Online database searches generated 10,907 records. Seven unique studies are included, combined with insight analysis from directed discussions with international advisors with lived experience of anxiety and/or depression and therapy (N = 8; mean age = 20.8), and mental health academics/clinicians (N = 6).

**Results:**

Findings are presented as a narrative synthesis and suggest that goal setting is helpful to young people experiencing anxiety and/or depression because it helps build good therapeutic relationships through open communication and building trust. Goal setting helps make things more manageable, enabling young people to feel supported and have ownership of their care. Individual preferences, or high levels of distress, trauma, low confidence, hopelessness, negative past experiences of goal setting, perfectionism, and rumination are considered limiting factors to goal setting. Additionally, contextual factors including country and long-term therapy are explored.

**Conclusion:**

Whilst the resultant sample is small, emphasis on the voices of young people in the research is both prominent and of paramount importance. Several key literature gaps are identified, including evidenced links to the reduction in symptoms. Priority must be given to researching unhelpful mechanisms of goal setting for young people experiencing anxiety and/or depression, to avoid any potential iatrogenic effects.

**Supplementary Information:**

The online version contains supplementary material available at 10.1186/s40359-022-00879-5.

## Background

Collaborative goal setting within therapeutic mental health settings refers to agreements made between young people and practitioners about specific therapy areas of focus: topics of personalised and meaningful outcome. Goals are concrete representations of intended endpoints, which fill the perceived gap between the current and desired end state [1]. Goals are usually formulated at the start of therapeutic interventions through a series of discussions. These differ from academic, physical rehabilitation, or general life goals, although there could be overlap. Progress towards these agreed goals may then be tracked over time, often through ratings on numerical scales, and there are tools available to support this. For example, the Goal Based Outcome tool (GBO; [2]) which comprises setting up to three goals and scoring progress between 0 and 10, is widely used to track progress against goal setting in youth mental health settings. Whilst goal tracking may lead to a shift in practitioners’ work to be goal focused [3], goals may also sit alongside usual clinical work, to track progress [4]. Goals set in therapy tend to be focused and specific, e.g., to deal with something in the immediacy, like a phobia [5], but it is important that these goals attain to more global goals [6], or are viewed as a “means to an end”.

Goals may take time to set, and can change and become more specific during the therapeutic process, for example, at the beginning of contact with a practitioner, a young person might have a general goal like “to feel less depressed”, but over time the young person, along with the practitioner, may learn more about the mechanisms behind the depression and may define more precise goals like “being able to stop negative thinking” or “being able to cope with flashbacks”. The types of phrases used by practitioners to help young people define goals may include: “what do you want to be different?”, “what will you get off your back?”, “where do you want to get to?”, and “how do you want things to change?” [4, 6].

Goal setting and tracking in therapeutic settings is grounded in motivation theory [7–9] such that working towards goals is a continuous feedback loop which builds on self-efficacy, self-determination and motivation to continue to strive towards goals, acting as a self-regulation strategy [10, 11]. Goal setting may be more feasible or acceptable to individuals with particular personality traits e.g., individuals who attribute successes and failures to external factors are less likely to find meaning in striving towards goals than those who attribute successes and failures to their own actions [12].

Further, young people have described recovery from depression as nested within relationships (e.g., [13]), portraying recovery as an intentional process, contingent on shared goals and joint action in relationships [14]. Good therapeutic relationships are considered a key element of effective therapy [15–18]. Also known as working relationships, or working/therapeutic alliance, this refers to the connection, bond or partnership between the young person and practitioner. Three key elements of therapeutic alliance have been identified in the literature: bond, tasks, and goals [19]. In a recent review of the effects of cognitive behavioural therapy (CBT) for young people experiencing anxiety and/or depression, three studies reported small-to-medium effect sizes for the correlational relationship between therapeutic alliance and symptom reduction [20]. This provides limited evidence linking goal collaboration to reduced anxiety/depression symptoms for young people, despite fair evidence supporting links between goal collaboration and positive adult anxiety and depression outcomes [21]. It is argued that goal agreement is a fundamental element missing from much work with young people, and it has been referred to as a “social contract” [22]. This emphasis on relationships is particularly important when working with young people with acute, or multifarious difficulties, where relationships are complex, difficult to develop and maintain (e.g., [23]).

Existing evidence suggests that there are certain elements of mental health support for young people that are effective, but there is a lack of identification and knowledge about mechanisms to refine and improve this support [24]. Specifically, there is a paucity of research exploring the mechanisms underpinning why goal setting may be helpful for some young people, and not others. There are likely to be confounding variables which interplay the effectiveness of goals, depression and/or anxiety, cognition, and motivation, yet there is little research that has explored this in clinical settings with young people.

The aim of this study is to summarise existing literature, supplemented by discussions with international advisors to contextualise and aid interpretation of the findings. The research question is:

“Is collaborative goal setting helpful or unhelpful to young people experiencing anxiety and/or depression, as an element of therapeutic relationships?

- Why/why not and how?
- For whom?
- Under what circumstances?”

## Methods

A mixed methodological approach combined reviews of peer-reviewed, grey literature and additional sources (e.g., websites), with consultation with experts by experience. The risk of expert view biasing the findings was mitigated via the validating steps outlined below. The study was designed by the lead researcher, and other researchers in the team, in collaboration with the peer researchers.

Whilst it is acknowledged that there are important outcome areas such as quality of life and existential factors, aside from symptom reduction, the focus of this study was to specifically explore the research questions in relation to potential anxiety and depression symptom reduction. Anxiety and depression were focused on as the most common mental health difficulties worldwide. This focus on medicalised symptomology differs from quality of life, which is a multi-dimensional construct comprised of several domains, such as psychological, physical, and social wellbeing. Anxiety, depression, therapeutic relationships, and goal progress are routinely measured using self- and proxy-reported outcome measures, with numerical rating scales. It was anticipated that the research question would not be adequately explored through findings from outcome measures alone. Based on some initial scoping work, we determined that there would be more evidence on the effectiveness of goal setting and tracking via qualitative enquiry, including narratives. The exploration of the nuances identified in the research question was key to the study, and so it was important to give precedence to young people’s voices through existing research and youth advisors, combined with findings from any relevant supporting measures. Such explorations would not be possible through quantitative enquiry of outcome measure data.

Goal setting alongside usual clinical work and goals work (goal focused interventions) were differentiated from implicit goal-oriented practice, non-directive approaches and paternalistic approaches to support in this study. This meant that to be included in the literature synthesis, goals needed to be explicitly identified as an approach to progress tracking, and/or informing the work. This study also focused on individual settings, and whilst these relationships may include parents/carers in a triad, the primary focus was on the relationship built between the practitioner and the young person. This was due to the complexities and potential dilution of agreeing goals and developing therapeutic relationships in group work and with parents/carers in addition. Ethical approval was not required because this study did not involve collection nor analysis of primary data, and youth advisors were consulted on in the capacity of being part of the advisory group, rather than within the capacity of research participants [25].

### Literature review

First, search terms and inclusion and exclusion criteria were agreed in collaboration with the academic/clinical and youth advisors (See Additional file 1: Appendix 1 Inclusion and exclusion criteria and Search Strategies). The project was registered with PROSPERO (number: CRD42021259611).

Second, searches of ten online databases were conducted (PsycINFO (OVID), MEDLINE (OVID), EMBASE (OVID), Web of Science core collection, current contents connect, SciELOCitation Index, Cochrane Library of Systematic Reviews, CINAHL (EBSCO), ERIC (EBSCO), and child and adolescent studies (EBSCO)). The search strategy developed for each database comprised three concepts: anxiety and/or depression (condition), goals (intervention) and therapeutic alliance or general views on goal setting, e.g., perspective, view, narrative (intervention/outcome). Searches were restricted to the past 20 years (2000-present). Citation tracking of included papers was performed. Retrieved hits were exported to EndNote 20 [26], Rayyan [27] and Excel for title/abstract screening.

Third, two researchers (FS, JJ) independently screened titles and abstracts. Where one researcher (JJ) was an author in retrieved studies, screening was conducted by the other researcher (FS), to ensure unbiased screening. Fourth, two researchers (JJ, IS) explored resultant literature main texts, extracting and synthesising relevant information. Key literature identified by researchers and advisors was added. The quality of the studies was assessed using criteria for qualitative studies ([28]; See Additional file 1: Appendix 2 Core Criteria for Quality Assessment of Qualitative Studies).

### Grey literature search

Google and Google Scholar title search, Google Books, PsycEXTRA, PsyArXiv, and ProQuest Dissertations and Theses were used. Google's Site Search was used to search American Psychological Association, British Psychological Society, Australian Psychological Society, European Federation of Psychologists' Associations, International Association of Applied Psychology, Association for Psychological Science, International Union of Psychological Science, Canadian Psychological Association, and UN-affiliated websites (.int domains). To identify more relevant literature, ResearchRabbit.ai was used to track the citations to the included studies. As a result of Google title search, websites were identified and browsed. The searches were restricted to those: (1) written in English, (2) published from January 2000 to August 2021, (3) focused on goal setting with young people experiencing mental health difficulties. Two researchers (FS, JJ) independently screened titles and abstracts of the resultant sources for relevance.

### Insight analysis

An advisory group was formed at the study’s outset, comprising: (1) young people with lived experience of anxiety and/or depression and therapy (N = 8; age range 15–26 years; mean age = 20.8; female (includes transgender) N = 5; and male (includes transgender) N = 3; located in Brazil, Pakistan, Spain, Turkey, and UK); and (2) academics and clinicians (N = 6; female N = 1, male N = 5; located in Norway and UK). Criteria for youth advisors to take part where that they were around the age of interest (14–24 years) and had lived experience of anxiety and/or depression and had previously -or currently-experienced receiving a mental health intervention. Youth advisors’ experience of anxiety and/or depression was balanced across advisors. Youth advisors were recruited via adverts circulated by a European network of peer advisors with international reach, and signed an agreement at the outset of the project, by way of consent to participate, which included specific duties and responsibilities of what would be expected of them, as well as hours and reimbursement details. For those under 18 years old, parent/carer consent and agreements were gained. One-to-one meetings between each youth advisor and the participation lead for the study were conducted before and after the study took place. A written agreement was made between the lead research organisation, and the participation organisation which facilitates the network of peer advisors.

Academic/clinical advisors were experienced and specialised in goals work and were recruited via existing networks. Criteria for academic/clinical advisors were that they had research and/or clinical experience in the field of mental health goal setting with young people (academic N = 6; clinical N = 4; categories not mutually exclusive). Written agreements were made between the lead research organisation, and each academic/clinical advisor.

Directed discussions were held at six advisory group meetings (two academic/clinical and four youth) facilitated by two researchers (JJ, MS) and conducted in English. All advisors spoke English, but time was given in the meetings to check understanding, as English was not a native language for many. The academic/clinical and youth advisors met separately, enabling the youth advisors to share openly with their peers. These discussions focused on the research question and drawing inferences about resultant findings, as well as appraising the evidence to identify key literature gaps. The summary of findings from the literature review was presented via PowerPoint to the advisors. The questions asked were broadly: is setting goals an important part of the relationship with the therapist and why/not; do these findings align with your experiences; is there anything you can think of that has not been considered; are there any elements of these findings that do not make sense in your experience; how do you interpret and understand these findings within the context of your own experience? Youth advisors were asked additional questions about the nature of language, for example, what do you think about the term “goal”? Is it the word you use, is it understandable, how does it translate to your national languages?. Field notes were taken, alongside notes in advisors’ own words on the JamBoard interactive workspace, allowing for anonymous contributions. Analysis comprised four stages. First, one researcher (MS) organised field notes and comments into a narrative summary. Second, one researcher (JJ) used the nuanced elements of the research question to organise the summary. Third, feedback was sought from advisors to evaluate and assess whether it was a true reflection of the discussions. Fourth, one researcher (JJ) refined and renamed the themes.

## Results

**Fig. 1:**
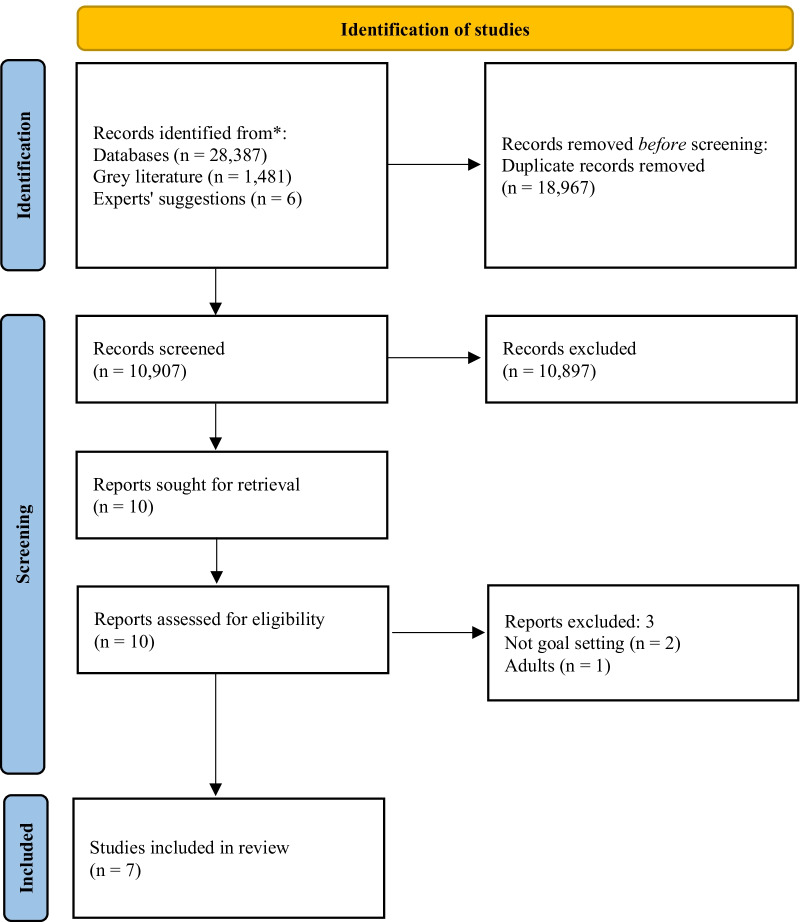
PRISMA flow chart of the study selection process. *From:* Page, M.J., McKenzie, J.E., Bossuyt, P.M., Boutron, I., Hoffmann, T.C., Mulrow, C.D. et al. The PRISMA 2020 statement: an updated guideline for reporting systematic reviews. BMJ. 2021;372(n71)

Online searches generated 10,907 records. Ten potentially eligible studies were identified. Upon screening full texts, seven unique studies met the selection criteria (See Fig. 1 and Table 1).

**Table 1:** Evidence synthesis of included studies (N = 7)

References	Participants, study type and location	Findings	Strengths and limitations of study	Critical appraisal of study*
Bennett LR. Adolescent Depression: Meeting Therapeutic Challenges Through an Integrated Narrative Approach. J Child Adolesc Psychiatr Nurs. 2012;25(4):184–94	N = 1 Male 14 years old Ethnicity: not reported	Collaborative goal setting influences therapeutic alliance via shared understanding of the difficulties, from the practitioner’s perspective. Goal setting is facilitative of good communication	Voice is given to the young person through the research, however, the study is primarily from the practitioner’s perspective	*Credibility:* some evidence (verbatim quotes)
	Major depressive episode with anxiety		Single case example	*Transferability:* caution advised when applying to other contexts
	Narrative case study	Emphasis on flexible working, via goals, to meet personal needs	Full demographics not reported	*Dependability:* not evidenced
	Canada		Western high-income country	*Confirmability:* not evidenced
Berdondini L, Elliott R, Shearer J. Collaboration in Experiential Therapy. J Clin Psychol. 2012;68(2):159–67	N = 1 Male 20 years old Ethnicity: European	Notes that there is no alliance without collaboration. Collaboration (goals and tasks) should be agreed with the client to ensure (1) Optimal working with their experiences; (2) Autonomy and acceptance of responsibility for own experiences/actions; (3) Completion of important therapeutic work	Voice is given to the young person through the research, however, the study is primarily from the practitioner’s perspective	*Credibility:* not evidenced
	Social anxiety	Case showed that productive collaboration is not necessarily natural or spontaneous but requires efforts of both the young person and practitioner to take emotional/interpersonal risks by disclosure of a genuine manner	Single case example	*Transferability:* caution advised when applying to other contexts
	Narrative case study		Full demographics not reported	*Dependability:* not evidenced
	UK	Goals are a conduit for good communication and increase engagement	Western high-income country	*Confirmability:* not evidenced
Cirasola A, Midgley N, Fonagy P, Martin P, M Goodyer I, Reynolds S, et al. The factor structure of the Working Alliance Inventory short-form in youth psychotherapy: an empirical investigation. Psychother Res [Internet]. 2021;31(4):535–47	N = 465 11–17 years (M = 15.5) Ethnicity: Majority White British (~ 74%)	Findings suggest that agreement on goals may be challenging in an age group who are often referred for therapy by others, and may not see difficulties “in” themselves, and developmental needs may interfere with the establishment of collaborative relationships with adults	Therapeutic alliance was rated over several time points	*Credibility:* good evidence
	Depression		Focus of the study was young people’s ratings on a therapeutic alliance tool (WAI-S)	*Transferability:* caution advised when applying to other contexts
	Randomised control trial assessing the effects of three therapeutic interventions in the treatment of adolescent depression	High levels of collaboration (goals and tasks) foster the development of a strong bond	Participants recruited from multiple settings	*Dependability:* some evidence
			Unable to control for variance at the client or practitioner level	*Confirmability:* not evidenced
	UK		Western high-income country	
Diamond GM, Diamond GS, Liddle HA. The therapist–parent alliance in family‐based therapy for adolescents. J Clin Psychol. 2000;56(8):1037–50	N = 1 Male 15 years old Ethnicity: White	Goal formulation is described as an important element of building therapeutic alliance. In the goal-formation phase, the practitioner aims to establish relationship building as the initial objective of therapy	Voice is given to the young person through the research, however, the study is primarily from the practitioner’s perspective	*Credibility:* some evidence (verbatim quotes)
	Depression		Single case example	*Transferability:* caution advised when applying to other contexts
	Narrative case study	Findings suggest that through specific goal setting, it is possible to increase disclosure, the sharing of vulnerable emotions, and mutual support between parent – young person – practitioner triads, during later conjoint conversations about difficult, relational themes	Western high-income country	*Dependability:* not evidenced
	Israel	Goal setting with parents/carers can facilitate open communication with young people		*Confirmability:* not evidenced
Feltham A, Martin K, Walker L, Harris L. Using goals in therapy: The perspective of people with lived experience. In: M. Cooper & DL, editor. Working with goals in psychotherapy and counselling. Oxford: Oxford University Press; 2018. p. 73–85	“Young people” or “young adults” age not defined	Agreeing goals in therapy is of key importance to young people, for communication: to enable them to feel heard, and that practitioners are listening to them	Narrative review incorporating quotes and examples from young people/young adults with lived experience enables important voices to be centralised in this research	*Credibility:* some evidence (verbatim quotes)
	Anxiety and depression are discussed as well as other difficulties			*Transferability:* caution advised when applying to other contexts
	Narrative review of people with lived experience of mental health difficulties and therapeutic interventions	Low confidence or feelings of hopelessness and poor previous experiences of goal setting are limiting factors for prospective goal setting	Full demographics not reported	*Dependability:* not evidenced
	UK		Western high-income country	*Confirmability:* some evidence
Law D, Wolpert M. Guide to using outcomes and feedback tools with children, young people and families. Formally known as the COOP document. London: CAMHS Press; 2014	“Young people” or “young adults” age not defined	Discusses the importance of building good therapeutic relationships, and cites goals as a central element of this. Provides quotes from young people based on their experiences of setting goals in therapy	Guidance for practitioners based on focus groups with practitioners and young people, incorporating quotes and examples from young people with lived experience enables important voices to be centralised in this research	*Credibility:* some evidence (verbatim quotes)
	Anxiety and depression are discussed as well as other difficulties			*Transferability:* caution advised when applying to other contexts
	Guidance document for practitioners for using feedback and outcome tools with children and young people in therapeutic settings	Reviewing progress towards goals too frequently in long-term therapy could give the young person the impression that the practitioner is more interested in gauging their own success, rather than being interested in them as a whole person	Full demographics not reported	*Dependability:* not evidenced
	UK	Young people in long-term therapy may have difficulty engaging in goal setting. This is due to the potential of having experienced significant and repeated traumas which have impacted on their development	Western high-income country	*Confirmability:* not evidenced
Martin K. A critical realist study of shared decision-making in young people’s mental health inpatient units. 2019. Doctoral Thesis. UCL	N = 16 Female N = 10 Male N = 5 Gender fluid N = 1 15–17 years (M = 16.2) Ethnicity: Bangladeshi British N = 1 Black African N = 1 Black British N = 1 British Romanian N = 1 Latino N = 1 White British N = 10 White Other N = 1	Findings emphasise the importance of building good therapeutic alliances and this study cites goals as a central element of shared decision making. The findings include quotes from young people based on their experiences of setting goals within the inpatient setting	The focus on qualitative interviews with young people enables important voices to be centralised in this research	*Credibility:* some evidence (verbatim quotes)
	Anxiety and depression and several other mental health difficulties		Sample recruited from two settings	*Transferability:* caution advised when applying to other contexts
	Naturalistic study based in two inpatient mental health units	The importance of a collaborative way of setting goals was expressed as a key element of ensuring goals is an effective element of helping, such that the collaboration of the practitioner is key to ensure the young person is setting achievable goals. Goals are a facilitator of good communication and a shared understanding of ways forward	Primarily White British sample and study conducted in a Western high-income country	*Dependability:* good evidence
	UK	Receiving feedback on progress towards goals is seen as a key motivator and provides validation that goals are meaningful and achievable		*Confirmability:* good evidence

*Definitions drawn from Hannes K. Chapter 4: Critical appraisal of qualitative research. In: Noyes J, Booth A, Hannes K, Harden A, Harris J, Lewin S, Lockwood C (editors), Supplementary Guidance for Inclusion of Qualitative Research in Cochrane Systematic Reviews of Interventions. Version 1 (updated August 2011). Cochrane Collaboration Qualitative Methods Group, 2011. See Additional file 1: Appendix 2 for detailed definitions of the appraisal criteria

Included studies comprised three narrative case studies [29–31] a randomised control trial [32]; a narrative review [33] a practitioners’ guidance document [34]; and a naturalistic study [35]. Critical appraisal of the evidence (Table 1) demonstrates that caution must be exercised when considering the findings. The main strength of the included studies is the voice of young people through verbatim quotes, and for some, strong consideration of the researchers’ impact. However, less strength is attributed to the dependability or generalisability of the findings, mainly due to the high proportion of small-and-homogenous samples. The advisors’ discussion summaries were organised into themes within the nuances of the research question: *Why/why not and how? For whom? Under what circumstances?”,* and presented as a narrative synthesis.

### Why/why not and how (mechanisms)

#### A conduit for open communication

Six studies described collaborative goal setting as a conduit for communication [29–31, 33–35]. Specifically, agreement on goals leads to open communication, a shared understanding of difficulties and ways forward [29, 31, 35]. Formulating goals was described as key to helping young people to feeling understood, valued and that practitioners are listening to them [33–35]. Collaborative goal setting enables young people and practitioners to make genuine disclosures, not necessarily otherwise possible [30] and facilitates mutual support [31].

Both academic/clinical and youth advisors said that open communication and trust were key, broadly agreeing that goal setting could be helpful to support building trusting relationships. It was agreed that collaboratively agreeing goals may take time and should not happen immediately. Rather, practitioners should work flexibly, aiming to understand what is comfortable for young people experiencing anxiety and/or depression. Some youth advisors said that relationships need to be built first, with trust established prior to goal setting, particularly when goal setting feels complicated. It was agreed by youth and academic/clinical advisors that goal setting should be led by young people and guided by practitioners, sharing responsibility. Youth advisors considered open communication the most crucial factor in therapy, with a sense that much therapeutic work cannot take place without it.

#### Feel supported and involved

Young people value receiving support to split actions into smaller manageable steps, with encouragement from practitioners stimulating validation that their goals are achievable ([35], and youth advisors). Being given choice about goal content and how this translates into the options for care was identified as an important part of the process in the literature [35]. Evidence suggests that this leads to a sense of autonomy and control over what happens to young people and enables them to feel involved in the process and increases engagement [30, 33, 35]. This was not directly addressed by the academic/clinical advisors in their discussions.

### For whom

#### Nature of difficulties

All seven studies, and youth and academic/clinical advisors, suggested that goal setting was a helpful element of therapeutic relationships for young people experiencing anxiety and/or depression, and more broadly with other undefined presenting difficulties. Both academic/clinical and youth advisors agreed that there was no need to separate specific attributes of anxiety or depression, due in part, to high proportions of comorbidity.

#### Age, and previous experiences

Three studies described difficulties for young people engaging in goal setting [32–34]. These were: age-appropriate quests for independence interfering with establishing collaborative relationships with adults [32]; significant and repeated traumas impacting development, relationships and challenges ordering thoughts, particularly within the context of long-term therapy [34]; low confidence or feelings of hopelessness; and poor previous experiences of goal setting [33]. Youth advisors agreed that previous life experiences were important, e.g., views of goal setting in therapeutic settings were impacted by how successful they had been in achieving past goals, regardless of goal type. Academic/clinical advisors agreed that personal factors such as previous experiences and factors surrounding—or leading to—difficulties, may lead to challenges setting goals in the first instance.

#### Levels of distress, personality traits and preferences

Youth and academic/clinical advisors suggested that specific unhelpful elements may depend on the young person, and sometimes levels of distress, rather than the nature of difficulties. Some youth advisors expressed preferences for practitioner-directed work, particularly in times of high distress, e.g.,: *“If I’m going through something very bad, I can be very frustrated/sad so I can’t think clear”* (youth advisor)*.* It was also agreed that goals may exacerbate anxiety, particularly at times of overwhelm, whilst for others this could be a helpful anxiety reduction approach, e.g., in exposure therapy. Youth advisors said that ensuring goals are achievable is key to building good therapeutic relationships, and the impact on anxiety/depression; the individual’s capacity to set goals should be considered, e.g., someone struggling with day-to-day tasks may find even small goals too challenging. Youth advisors considered perfectionism to be important, where some people may feel pressure to *achieve* goals. A sense of hopelessness, or procrastination, and rumination also, where delaying tasks may result in delaying work on goals. For some youth advisors, goal setting felt especially important, whilst for others it was not, rather a supportive relationship was identified as most important, and they could not see how that would be developed through goal setting. Academic/clinical advisors said that young people’s preferences to work on goals, or not, was in itself of key importance to the therapeutic relationship. There was no evidence from the included literature to support/oppose these points.

#### Language and power dynamics

Linked to preferences, youth advisors said that young people tend not to like the term “goal” because they attribute it to work and formal settings, whereas “therapeutic goals” are personal with deeper meaning. Academic/clinical advisors discussed using alternative language for goal setting and goal directed work, and the importance of being led by the young person. Posing questions such as “What do you want to change?” is suggested as an alternative in the literature ([33]; p.47). Youth advisors said that whilst some young people may feel able to say they do not want to set goals, others may not, due to the young person-practitioner power imbalance, which has implications for relationships, and therapeutic work. There was no further evidence from the included literature to support/oppose these points.

### Under what circumstances (contextual factors)

#### Broadly helpful

All seven studies suggested that goal setting was a helpful element of therapeutic relationships for young people within the research contexts. This included year-long narrative therapy with interpersonal therapy and CBT techniques in alliance with the family [29]; multimodal family therapy [31]; Gestalt therapy [30]; either CBT, short-term psychoanalytic psychotherapy or brief psychosocial intervention [32]; UK child and adolescent mental health services [33, 34] and UK inpatient settings [35]. All studies were based in Western high-income countries. Academic/clinical and youth advisors agreed with this assessment.

#### Review points and referral routes

Reviewing progress towards goals too frequently could give the impression that practitioners are more interested in gauging their own success, rather than in the young person as a whole person, and rating could end up being done by rote, making goals increasingly meaningless [34]*.* Academic/clinical and youth advisors agreed with this, discussing the need to work with goals in a flexible manner. Additionally, young people may not recognise the symptoms identified, particularly when referred for treatment by another party (e.g., parents/carers), which is crucial to enable collaborative goal setting [32]. Challenges associated with thinking of goals in this way was addressed by the academic/clinical and youth advisors in wider discussions elsewhere (see therapy contexts).

#### Culture and therapy contexts

Youth and academic/clinical advisors located in Western high-income countries agreed that it may depend on types of interventions offered and practitioner’s preferred working style, but young people largely have agency to set goals. However, it was recognised by the youth and academic/clinical advisors that some young people in some countries do not have agency to set goals. There, decisions are made by families, in collaboration with practitioners, and so less consideration is given to young people’s perspectives. It was suggested that, in some countries, there is no concept of setting goals (e.g., a youth advisor discussed their experience in Pakistan), and ongoing stigma associated with mental health difficulties, which may lead to distrust, scepticism in, and a disconnect with practitioners. Youth advisors said that this may also be true in other countries not represented. A youth advisor suggested that young people in Brazil were relaxed towards goal setting and would not mind if goals were not achieved; directed therapy was considered more helpful.

Youth and academic/clinical advisors discussed goals in long-term therapy as potentially feeling restrictive, with challenges associated with thinking of what goals might be. Both long-and short-term goal setting within this context may feel meaningless, which if then pressed by the practitioner, has a negative impact on relationships. Academic/clinical advisors said that the feasibility of goal setting in the first instance is likely to be attributable to the factors young people who might be offered long-term therapy might have, rather than the work itself leading to these challenges. Youth and academic/clinical advisors also said that where there are multiple needs and risks, goals need to be simpler to feel manageable. Youth advisors said that sometimes there were concerns about the achievement of goals equating to treatment ending, which felt unsettling. There was no evidence from the included literature to support/oppose these points.

## Discussion

This study aimed to provide a synthesis of existing literature, identifying knowledge gaps. Whilst much may be drawn from related research, caution must be exercised when translating findings into other contexts [11], and whilst promising, generalising adult findings to youth must be exercised with an abundance of caution. Evidence suggests that adults and children think differently; as children grow, their cognitive processes develop, and their contexts and perspectives change, impacting on understandings of the self and the world around them. Further, models of recovery from depression are notably different between adults and young people [14]. As such, we have focused on evidence from the youth field in our discussion, and further highlight the paucity of research with young people in this area.

The included evidence originates from Western high-income and largely specialist settings; further research in majority world countries is urgently required. Many studies identified in initial searches only partially met inclusion criteria. This evidence paucity may suggest goal setting is not embedded in service standards or practice in most countries, or other limiting factors such as the general underfunding of youth mental health research. Some examples were derived from the insight analysis, highlighting the advisors’ value, who helped contextualise and interpret evidence, grounded in lived experience. However, whilst the research question pertained to the effectiveness of goal setting as part of therapeutic relationships, the findings were related to the feasibility, or acceptability of goal setting itself. Links between effective goal setting, good therapeutic relationships and positive outcomes are inferred based on evidence that partially supports the research question, and the discussions with the advisory group, but no evidence relating to anxiety or depression outcomes was found in this study. Future research should consider in depth explorations of mechanisms of goal setting within therapeutic relationships, for young people experiencing anxiety and/or depression.

For many young people, goal setting is a helpful tool for building good therapeutic relationships via open communication. These findings support previous research which partially address the research question: young people find goal setting to be helpful to therapeutic relationships through the development of a shared language and understanding [3]. It has been suggested that goals are a mechanism of change via a means for “common ground” to be established [3]. Finding common ground and a shared understanding are particularly pertinent in youth mental health settings, where there are multiple stakeholders involved [36–38], which can be a balancing act [39]. Establishing this mutuality of situations is considered the key facilitator of engagement when referred for therapy by others [40]. Further, ownership of goals located with young people is important [41], which in turn gives young people ownership of their care, which can be motivational [42, 43]. Young people experiencing anxiety may find goal setting an effective strategy due to links with avoidance motivation; such that they have reported pursuing approach goals to avoid negative emotional consequences of not doing so [44]. The ability of young people to maintain focus on the pursuit of personal goals has also been demonstrated as a moderator of depression and suicide [45].

One included study explicitly discussed parents/carers within collaborative goals and therapeutic relationships, as a foundation for mutual support [31]. Stronger relationships between both young people, parents/carers and practitioners and/or involving both young people and parents/carers in decision-making have been demonstrated to predict more positive outcomes [39, 46]. Young people are often referred by their parents/carers, which must be considered, particularly where literature highlights challenges of setting goals when young people do not agree with the referral or recognise the difficulties [22, 32]. Prior research has demonstrated that young people from minoritized ethnic groups are more likely to be referred for mental health support via social care and the youth justice system compared to their White British counterparts, who are commonly referred via primary care in the UK [47]. Further, evidence suggests that increases in emotional autonomy result in a shift from dependence on adults in adolescence, to reliance upon peers for support [48] particularly amongst girls [49], which may align with the developmental interference with building relationships outside of goal setting found by Cirasola and colleagues [32]. It has been argued that for young people who have difficulties building and maintaining relationships, the therapeutic relationship is particularly important (e.g., [23]). It is also noteworthy that young people in some countries may not have agency to set goals, a significant limiting factor. There are cultural and service level factors which were not explored. In some cultures, advice is sought from family and religious leaders over mental health professionals (e.g., [50]). Organisational level factors have also been found to hinder and influence therapeutic processes [40]. Further research is needed into referral routes, and intersections between systems, practice, and young people’s preferences.

Several elements of goal setting were identified as unhelpful for young people experiencing anxiety and/or depression, supporting previous literature. These discussions centred on the feasibility/acceptability of goals, rather than goal setting being detrimental to therapeutic relationships per se. Nevertheless, it is suggested that these factors were primarily related to the person, and that “personal” factors may be driven by underlying difficulties. For example, low confidence, hopelessness, levels of distress, perfectionism, and rumination (e.g., [51–55], may all be elements of anxiety and/or depression. Academic/clinical and youth advisors agreed that goals may become clearer over time, particularly for young people experiencing depression and purposeless, and through collaboration, goals could be formulated. The importance of considering specific challenges of goal setting during long-term therapy was highlighted. Academic/clinical and youth advisors discussed challenges associated with identifying priority areas for work, and that goals continue to flex and change, with the potential for goals work to feel too restrictive. This is in support of previous research suggesting that it is important that goals are worked on flexibly [3] with space for them to change; specifically in relation to depression. Compared to those with low levels of depression, young people with high levels of depression are more able to disengage with unhelpful goals over time and to set new goals, which in turn may predict lower levels of depressive symptoms over a year later [56]. This sense of goals flexing, feeling unique and changeable has been mirrored in descriptions of therapeutic relationships themselves [23]. There was a clear steer from youth advisors that the relationship independent of goal setting was key to good outcomes, and that this was a priority; that without the trusting relationship, there is no facilitator for goal setting. This is an important contradiction to the literature, warranting further exploration. One suggestion is that the initial goals for long-term therapy should be on relationship building, but reviewed, so the therapeutic relationship itself does not remain the primary goal [34]. Another key finding is that goals take time to establish, and pressure to set goals may render them meaningless, which also supports previous research [51]. Young people often do not know what their goals are [57], which impacts trust building, relationships and thus, therapeutic work. In support of prior research which defines recovery as contingent on shared goals and joint action in relationships [14], links found between goals, trust building and therapeutic relationships in the present study align with research on trauma informed care, and emotional and relational safety (see, [58]). Further consideration should be given to this area, particularly clinical implications, and interactions with levels of distress.

Whilst support approaches that incorporate structured goal setting are often characterised by a greater emphasis on client-centredness, the links between personally meaningful outcomes and the specific behaviour change techniques required to progress towards goals are not clear. Further, the person-centred focus is hypothesised as a conduit to positive ratings of self-efficacy, quality of life and service satisfaction, but evidence is lacking [11]. Whilst previous literature from within the youth mental health field suggests that working on goals is motivating and increases self-efficacy [34, 42], evidence is still limited. Goal setting may be useful to young people because, whilst not necessarily synonymous, it has been demonstrated as a facilitative element of shared decision-making [59, 60]. This collaborative way of working through shared understanding and the development of good therapeutic relationships [61] may be especially helpful to young people experiencing depression as it enables them to exercise control over their own feelings and behaviour [43] at a time when they may be experiencing feelings of hopelessness and purposeless. Whilst educated links are made to shared decision-making, further research should explore whether there is an embedded link to goals and therapeutic relationships.

### Strengths and limitations

The mixed-methodological approach was a particular strength, with literature findings bolstered by lived experience. However, whilst advisors were from diverse demographic groups, not all groups were represented.

Whilst every attempt was made to include as many goal setting search terms as possible, the language is broad and fluid, meaning certain terms may have been missed. Still, the high number of results returned from literature searches suggests the strategy may need refinement. Nevertheless, we chose to ensure a large return given the subject’s broad nature. At the screening stage, the focus on explicitly identified goal setting and goal work made the identification of included studies less ambiguous, but meant that studies focused on implicit goals work would not have been included, reducing the number of studies included in the final synthesis.

Prior assumptions and knowledge of this topic will have influenced the researchers’ interpretation of the findings, even subconsciously. This includes the decision to use the nuanced elements of the research question to organise the findings. The researchers were located in Belgium, Germany, and the UK at the time of the study, which risks the perpetuation of the status quo of Western high-income-originating dominated research. Further, the findings were contextualised and linked to prior theory primarily by a researcher outside the age range of interest (JJ). The impact of both issues was mitigated via advisors, particularly those within majority world countries and the age range of interest, and the peer researchers entrenched in the research team (MS, IS), who provided contextual depth and understanding to the findings.

## Conclusion

Literature focused on goal setting as helpful for young people with anxiety and/or depression is overwhelmingly supportive, but this leaves research gaps regarding in which ways, for whom and under what circumstances goal setting might be unhelpful. Priority must be given to researching unhelpful mechanisms of goal setting, to avoid potential iatrogenic effects. Accessibility could be improved through exploration of the intersections between systems/contexts (e.g., country), therapeutic practice (e.g., practitioner’s training/preferences) and young people’s preferences. Further research is also needed to explore mechanisms by which goal setting may help to reduce anxiety and/or depression symptoms, as well as other important areas of outcome, such as quality of life, using e.g., mediation analysis.

Scaling up in countries with well-developed systems could mean embedding goals in guidelines for anxiety and/or depression; in service specifications, including monitoring and reporting change mechanisms; staff training in consistency; and some interagency forums to align goal processes. For majority world countries with less developed systems, largely relying on non-specialist services e.g., NGOs, goals may be paradoxically more important for maximising limited resources. Despite nothing suggesting goal setting could not practically be scaled-up globally, cultural considerations may be a limiting factor in some places.

Preferences to not work on goals may be driven by the limiting factors identified, such as hopelessness or high distress. Practitioners should work through this first, reviewing the option to work on goals over time, respecting young people’s preferences. Flexibility is important, and ownership of goals located with young people is essential, particularly to those experiencing depression, enabling them to exercise control over their feelings and behaviour when they may be feeling hopeless and/or purposeless. Finally, there may be a unique opportunity for goals to facilitate work with young people experiencing high distress levels or who have experienced trauma, due to links to emotional and relational safety and building trusting relationships.

## Supplementary Information

**Additional file 1. Appendix 1.** Inclusion and exclusion criteria and Search Strategies. **Appendix 2** Core Criteria for Quality Assessment of Qualitative Studies.

## Data Availability

The datasets generated and/or analysed during the current study are not publicly available to protect the confidentiality of the small number of advisors, but may be available from the corresponding author’s organisation, on reasonable request.

## Abbreviations

CBT: Cognitive behavioural therapy
GBO: Goal based outcomes tool
UK: United Kingdom
