# Supplementary material for: Goal setting with young people for anxiety and depression: What works for whom in therapeutic relationships? A literature review and insight analysis
Source: BMC Psychol. 2022 Jul 13;10:171. doi: 10.1186/s40359-022-00879-5 (PMC9281142; doi:10.1186/s40359-022-00879-5)
Supplement: Supplementary file 1 — Additional file 1. Appendix 1. Inclusion and exclusion criteria and Search Strategies. Appendix 2 Core Criteria for Quality Assessment of Qualitative Studies. [file 40359_2022_879_MOESM1_ESM.pdf]

Supplementary materials

Appendix 1. Inclusion and exclusion criteria and Search Strategies

Table A.1. Inclusion and exclusion criteria

Key focus (PICO)	Inclusion	Exclusion
Young People (age range 14-24 years)*	Mean age within the specified range	Mean age outside of specified range
Intervention/Moderator (Goals)	Focus on goals; discussion of setting goals or goal-focused working	No focus on goals
Condition (Mental health)	Anxiety (as defined)<br>Depression (as defined)	Non-mental health.<br>Mental health not including anxiety and depression.
Outcome<br>(Therapeutic Alliance<br><br>Or<br>or general views on goal setting (e.g., perspective, view, narrative)).	Focus on the working relationship between practitioners and young people, especially as a conduit to better outcomes as defined by young people	No mention of working relationships, shared decision making etc.<br>No exploration of the benefits/disbenefits of goals
Study type	All study types will be considered, e.g., narrative reviews, commentaries, narrative case studies, RCTs, surveys, naturalistic studies	None.

*Search term was removed from the search terms due to anticipated limitations of accessing relevant literature, e.g., the term “young people” would not capture those in the upper end of the age range. Inclusion and exclusion criteria were applied at the filtering stage

Search Strategies

a) **MEDLINE**

((Depression or (Anxiety or Anxiety Disorders) or Mood Disorders or (psychological trauma or sexual trauma or stress disorders, post-traumatic or stress disorders, traumatic, acute) or Psychological Distress) and ((task* adj2 (behavio* or mental health)) or (target* adj2 (behavio* or mental health)) or (aim* adj2 (behavio* or mental health)) or ("personal project*" adj2 (behavio* or mental health)) or (striving* adj2 (behavio* or mental health)) or (plan* adj2 (behavio* or mental health)) or (goal* adj5 (set* or agree* or plan* or consensus or negotiat* or discuss* or propos* or develop* or formulat* or elaborat* or establish* or identif* or write or written or state* or specif* or construct* or manag* or direct* or orient* or attain* or achiev* or evaluat* or cent?red or focus* or track* or progress*))) and (perspective* or view* or percept* or narrative* or opinion* or barrier* or facilitator* or

strength* or weakness* or concern* or understand* or judg* or catalyst* or obstacle* or feedback* or outcome* or (therapeutic alliance or Decision Making, Shared or (communication or negotiating) or (Interaction* or Relation* or Partner* or Agreement* or Disagreement* or engag* or disengag* or rapport or collab*)))).ti,ab.
limit 23 to (english language and yr="2000 -Current")

b) PsycInfo

((Depression or (Anxiety or Anxiety Disorders) or Mood Disorders or (psychological trauma or sexual trauma or stress disorders, post-traumatic or stress disorders, traumatic, acute) or Psychological Distress) and ((task* adj2 (behavio* or mental health)) or (target* adj2 (behavio* or mental health)) or (aim* adj2 (behavio* or mental health)) or ("personal project*" adj2 (behavio* or mental health)) or (striving* adj2 (behavio* or mental health)) or (plan* adj2 (behavio* or mental health)) or (goal* adj5 (set* or agree* or plan* or consensus or negotiat* or discuss* or propos* or develop* or formulat* or elaborat* or establish* or identif* or write or written or state* or specif* or construct* or manag* or direct* or orient* or attain* or achiev* or evaluat* or cent?red or focus* or track* or progress*))) and (perspective* or view* or percept* or narrative* or opinion* or barrier* or facilitator* or strength* or weakness* or concern* or understand* or judg* or catalyst* or obstacle* or feedback* or outcome* or (therapeutic alliance or Decision Making, Shared or (communication or negotiating) or (Interaction* or Relation* or Partner* or Agreement* or Disagreement* or engag* or disengag* or rapport or collab*)))).ti,ab.
limit 2 to (english language and yr="2000 -Current")

c) EMBASE

((Depression or (Anxiety or Anxiety Disorders) or Mood Disorders or (psychological trauma or sexual trauma or stress disorders, post-traumatic or stress disorders, traumatic, acute) or Psychological Distress) and ((task* adj2 (behavio* or mental health)) or (target* adj2 (behavio* or mental health)) or (aim* adj2 (behavio* or mental health)) or ("personal project*" adj2 (behavio* or mental health)) or (striving* adj2 (behavio* or mental health)) or (plan* adj2 (behavio* or mental health)) or (goal* adj5 (set* or agree* or plan* or consensus or negotiat* or discuss* or propos* or develop* or formulat* or elaborat* or establish* or identif* or write or written or state* or specif* or construct* or manag* or direct* or orient* or attain* or achiev* or evaluat* or cent?red or focus* or track* or progress*))) and (perspective* or view* or percept* or narrative* or opinion* or barrier* or facilitator* or strength* or weakness* or concern* or understand* or judg* or catalyst* or obstacle* or feedback* or outcome* or (therapeutic alliance or Decision Making, Shared or (communication or negotiating) or (Interaction* or Relation* or Partner* or Agreement* or Disagreement* or engag* or disengag* or rapport or collab*)))).ti,ab.
limit 2 to (english language and yr="2000 -Current")

d) Web of Science Core Collection

TOPIC: ((Depression or (Anxiety or Anxiety Disorders) or Mood Disorders or (psychological trauma or sexual trauma or stress disorders, post-traumatic or stress disorders, traumatic, acute) or Psychological Distress)) Indexes=SCI-EXPANDED, SSCI, A&HCI, CPCI-S, CPCI-SSH, BKCI-S, KCI-SSH, ESCI, CCR-EXPANDED, IC Timespan=2000-2021) AND TOPIC: ((perspective* or view* or percept* or narrative* or opinion* or barrier* or facilitator* or strength* or weakness* or concern* or understand* or judg* or catalyst* or obstacle* or feedback* or outcome*) or (therapeutic alliance or Decision Making, Shared or

(communication or negotiating)) or (Interaction* or Relation* or Partner* or Agreement* or Disagreement* or engag* or disengag* or rapport or collab*)) Indexes=SCI-EXPANDED, SSCI, A&HCI, CPCI-S, CPCI-SSH, BKCI-S, BKCI-SSH, ESCI, CCR-EXPANDED, IC Timespan=2000-2021 AND (TOPIC: ((aim* NEAR/2 behavio*)) OR TOPIC: ((aim* NEAR/2 mental health)) Indexes=SCI-EXPANDED, SSCI, A&HCI, CPCI-S, CPCI-SSH, BKCI-S, BKCI-SSH, ESCI, CCR-EXPANDED, IC Timespan=2000-2021 OR TOPIC: ((target NEAR/2 behavio*)) OR TOPIC: ((target* NEAR/2 mental health)) Indexes=SCI-EXPANDED, SSCI, A&HCI, CPCI-S, CPCI-SSH, BKCI-S, BKCI-SSH, ESCI, CCR-EXPANDED, IC Timespan=2000-2021 OR TOPIC: ((task* NEAR/2 behavio*)) OR TOPIC: ((task* NEAR/2 mental health)) Indexes=SCI-EXPANDED, SSCI, A&HCI, CPCI-S, CPCI-SSH, BKCI-S, BKCI-SSH, ESCI, CCR-EXPANDED, IC Timespan=2000-2021 OR TOPIC: ((personal project NEAR/2 behavio*)) OR TOPIC: ((personal project NEAR/2 mental health)) Indexes=SCI-EXPANDED, SSCI, A&HCI, CPCI-S, CPCI-SSH, BKCI-S, BKCI-SSH, ESCI, CCR-EXPANDED, IC Timespan=2000-2021 OR TOPIC: ((plan NEAR/2 behavio*)) OR TOPIC: ((plan NEAR/2 mental health)) Indexes=SCI-EXPANDED, SSCI, A&HCI, CPCI-S, CPCI-SSH, BKCI-S, BKCI-SSH, ESCI, CCR-EXPANDED, IC Timespan=2000-2021 OR TOPIC: ((striving* NEAR/2 behavio*)) OR TOPIC: ((striving* NEAR/2 mental health)) Indexes=SCI-EXPANDED, SSCI, A&HCI, CPCI-S, CPCI-SSH, BKCI-S, BKCI-SSH, ESCI, CCR-EXPANDED, IC Timespan=2000-2021 OR TOPIC: ((goal* NEAR/5 track*)) OR TOPIC: ((goal* NEAR/5 progess*)) Indexes=SCI-EXPANDED, SSCI, A&HCI, CPCI-S, CPCI-SSH, BKCI-S, BKCI-SSH, ESCI, CCR-EXPANDED, IC Timespan=2000-2021 OR TOPIC: ((goal* NEAR/5 set*)) OR TOPIC: ((goal* NEAR/5 agree*)) OR TOPIC: ((goal* NEAR/5 plan*)) OR TOPIC: ((goal* NEAR/5 consensus)) OR TOPIC: ((goal* NEAR/5 negotiat*)) OR TOPIC: ((goal* NEAR/5 discuss*)) OR TOPIC: ((goal* NEAR/5 propos*)) OR TOPIC: ((goal* NEAR/5 develop*)) OR TOPIC: ((goal* NEAR/5 formulat*)) OR TOPIC: ((goal* NEAR/5 elaborat*)) OR TOPIC: ((goal* NEAR/5 establish*)) OR TOPIC: ((goal* NEAR/5 identif*)) OR TOPIC: ((goal* NEAR/5 write)) OR TOPIC: ((goal* NEAR/5 written)) OR TOPIC: ((goal* NEAR/5 state*)) OR TOPIC: ((goal* NEAR/5 specif*)) OR TOPIC: ((goal* NEAR/5 construct*)) OR TOPIC: ((goal* NEAR/5 manag*)) OR TOPIC: ((goal* NEAR/5 direct*)) OR TOPIC: ((goal* NEAR/5 orient*)) OR TOPIC: ((goal* NEAR/5 attain*)) OR TOPIC: ((goal* NEAR/5 achiev*)) OR TOPIC: ((goal* NEAR/5 evaluat*)) OR TOPIC: ((goal* NEAR/5 cent?red)) OR TOPIC: ((goal* NEAR/5 focus*)) Indexes=SCI-EXPANDED, SSCI, A&HCI, CPCI-S, CPCI-SSH, BKCI-S, BKCI-SSH, ESCI, CCR-EXPANDED, IC Timespan=2000-2021)

e) Cochrane Library of Systematic Reviews

(Depression or (Anxiety or Anxiety Disorders) or Mood Disorders or (psychological trauma or sexual trauma or stress disorders, post-traumatic or stress disorders, traumatic, acute) or Psychological Distress):ti,ab,kw AND ((perspective* or view* or percept* or narrative* or opinion* or barrier* or facilitator* or strength* or weakness* or concern* or understand* or judg* or catalyst* or obstacle* or feedback* or outcome*) or (therapeutic alliance or Decision Making, Shared or (communication or negotiating)) or (Interaction* or Relation* or Partner* or Agreement* or Disagreement* or engag* or disengag* or rapport or collab*)):ti,ab,kw AND ((task or target or aim* or "personal project" or striving* or plan* or goal*)):ti,ab,kw

f) ERIC

AB ((Depression or (Anxiety or Anxiety Disorders) or Mood Disorders or (psychological trauma or sexual trauma or stress disorders, post-traumatic or stress disorders, traumatic, acute) or Psychological Distress) and (perspective* or view* or percept* or narrative* or opinion* or barrier* or facilitator* or strength* or weakness* or concern* or understand* or judg* or catalyst* or obstacle* or feedback* or outcome* or (therapeutic alliance or Decision Making, Shared or (communication or negotiating) or (Interaction* or Relation* or Partner* or Agreement* or Disagreement* or engag* or disengag* or rapport or collab*)) and ((task* N2 (behavio* or mental health)) OR AB ((target* N2 (behavio* or mental health)) OR AB ((aim* N2 (behavio* or mental health)) OR AB (("personal project*" N2 (behavio* or mental health)) OR AB ((striving* N2 (behavio* or mental health)) OR AB ((plan* N2 (behavio* or mental health)) OR (goal* N5 (set* or agree* or plan* or consensus or negotiat* or discuss* or propos* or develop* or formulat* or elaborat* or establish* or identif* or write or written or state* or specif* or construct* or manag* or direct* or orient* or attain* or achiev* or evaluat* or cent?red or focus* or task* or progress*)).
Limiters - Date Published: 20000101-20210631
Expanders - Apply related words; Apply equivalent subjects
Search modes - Boolean/Phrase

g) CINAHL Plus

AB ((Depression or (Anxiety or Anxiety Disorders) or Mood Disorders or (psychological trauma or sexual trauma or stress disorders, post-traumatic or stress disorders, traumatic, acute) or Psychological Distress) and (perspective* or view* or percept* or narrative* or opinion* or barrier* or facilitator* or strength* or weakness* or concern* or understand* or judg* or catalyst* or obstacle* or feedback* or outcome* or (therapeutic alliance or Decision Making, Shared or (communication or negotiating) or (Interaction* or Relation* or Partner* or Agreement* or Disagreement* or engag* or disengag* or rapport or collab*)) and ((task* N2 (behavio* or mental health)) OR AB ((target* N2 (behavio* or mental health)) OR AB ((aim* N2 (behavio* or mental health)) OR AB (("personal project*" N2 (behavio* or mental health)) OR AB ((striving* N2 (behavio* or mental health)) OR AB ((plan* N2 (behavio* or mental health)) OR (goal* N5 (set* or agree* or plan* or consensus or negotiat* or discuss* or propos* or develop* or formulat* or elaborat* or establish* or identif* or write or written or state* or specif* or construct* or manag* or direct* or orient* or attain* or achiev* or evaluat* or cent?red or focus* or task* or progress*)).
Limiters - Date Published: 20000101-20210631
Expanders - Apply related words; Apply equivalent subjects
Search modes - Boolean/Phrase

h) Child Development & Adolescent Studies

AB ((Depression or (Anxiety or Anxiety Disorders) or Mood Disorders or (psychological trauma or sexual trauma or stress disorders, post-traumatic or stress disorders, traumatic, acute) or Psychological Distress) and (perspective* or view* or percept* or narrative* or opinion* or barrier* or facilitator* or strength* or weakness* or concern* or understand* or judg* or catalyst* or obstacle* or feedback* or outcome* or (therapeutic alliance or Decision Making, Shared or (communication or negotiating) or (Interaction* or Relation* or Partner* or Agreement* or Disagreement* or engag* or disengag* or rapport or collab*)) and ((task* N2 (behavio* or mental health)) OR AB ((target* N2 (behavio* or mental health)) OR AB ((aim* N2 (behavio* or mental health)) OR AB (("personal project*" N2 (behavio* or mental health)) OR AB ((striving* N2 (behavio* or mental health)) OR AB ((plan* N2 (behavio* or mental health)) OR (goal* N5 (set* or agree* or plan* or consensus or

negotiat* or discuss* or propos* or develop* or formulat* or elaborat* or establish* or identif* or write or written or state* or specif* or construct* or manag* or direct* or orient* or attain* or achiev* or evaluat* or cent?red or focus* or task* or progress*)).
Limiters - Date Published: 20000101-20210631
Expanders - Apply related words; Apply equivalent subjects
Search modes - Boolean/Phrase

i) Current Contents Connect

TOPIC: ((Depression or (Anxiety or Anxiety Disorders) or Mood Disorders or (psychological trauma or sexual trauma or stress disorders, post-traumatic or stress disorders, traumatic, acute) or Psychological Distress)) Indexes=SCI-EXPANDED, SSCI, A&HCI, CPCI-S, CPCI-SSH, BKCI-S, KCI-SSH, ESCI, CCR-EXPANDED, IC Timespan=2000-2021) AND TOPIC: ((perspective* or view* or percept* or narrative* or opinion* or barrier* or facilitator* or strength* or weakness* or concern* or understand* or judg* or catalyst* or obstacle* or feedback* or outcome*) or (therapeutic alliance or Decision Making, Shared or (communication or negotiating)) or (Interaction* or Relation* or Partner* or Agreement* or Disagreement* or engag* or disengag* or rapport or collab*)) Indexes=SCI-EXPANDED, SSCI, A&HCI, CPCI-S, CPCI-SSH, BKCI-S, BKCI-SSH, ESCI, CCR-EXPANDED, IC Timespan=2000-2021 AND (TOPIC: ((aim* NEAR/2 behavio*)) OR TOPIC: ((aim* NEAR/2 mental health)) Indexes=SCI-EXPANDED, SSCI, A&HCI, CPCI-S, CPCI-SSH, BKCI-S, BKCI-SSH, ESCI, CCR-EXPANDED, IC Timespan=2000-2021 OR TOPIC: ((target NEAR/2 behavio*)) OR TOPIC: ((target* NEAR/2 mental health)) Indexes=SCI-EXPANDED, SSCI, A&HCI, CPCI-S, CPCI-SSH, BKCI-S, BKCI-SSH, ESCI, CCR-EXPANDED, IC Timespan=2000-2021 OR TOPIC: ((task* NEAR/2 behavio*)) OR TOPIC: ((task* NEAR/2 mental health)) Indexes=SCI-EXPANDED, SSCI, A&HCI, CPCI-S, CPCI-SSH, BKCI-S, BKCI-SSH, ESCI, CCR-EXPANDED, IC Timespan=2000-2021 OR TOPIC: ((personal project NEAR/2 behavio*)) OR TOPIC: ((personal project NEAR/2 mental health)) Indexes=SCI-EXPANDED, SSCI, A&HCI, CPCI-S, CPCI-SSH, BKCI-S, BKCI-SSH, ESCI, CCR-EXPANDED, IC Timespan=2000-2021 OR TOPIC: ((plan NEAR/2 behavio*)) OR TOPIC: ((plan NEAR/2 mental health)) Indexes=SCI-EXPANDED, SSCI, A&HCI, CPCI-S, CPCI-SSH, BKCI-S, BKCI-SSH, ESCI, CCR-EXPANDED, IC Timespan=2000-2021 OR TOPIC: ((striving* NEAR/2 behavio*)) OR TOPIC: ((striving* NEAR/2 mental health)) Indexes=SCI-EXPANDED, SSCI, A&HCI, CPCI-S, CPCI-SSH, BKCI-S, BKCI-SSH, ESCI, CCR-EXPANDED, IC Timespan=2000-2021 OR TOPIC: ((goal* NEAR/5 track*)) OR TOPIC: ((goal* NEAR/5 progess*)) Indexes=SCI-EXPANDED, SSCI, A&HCI, CPCI-S, CPCI-SSH, BKCI-S, BKCI-SSH, ESCI, CCR-EXPANDED, IC Timespan=2000-2021 OR TOPIC: ((goal* NEAR/5 set*)) OR TOPIC: ((goal* NEAR/5 agree*)) OR TOPIC: ((goal* NEAR/5 plan*)) OR TOPIC: ((goal* NEAR/5 consensus)) OR TOPIC: ((goal* NEAR/5 negotiat*)) OR TOPIC: ((goal* NEAR/5 discuss*)) OR TOPIC: ((goal* NEAR/5 propos*)) OR TOPIC: ((goal* NEAR/5 develop*)) OR TOPIC: ((goal* NEAR/5 formulat*)) OR TOPIC: ((goal* NEAR/5 elaborat*)) OR TOPIC: ((goal* NEAR/5 establish*)) OR TOPIC: ((goal* NEAR/5 identif*)) OR TOPIC: ((goal* NEAR/5 write)) OR TOPIC: ((goal* NEAR/5 written)) OR TOPIC: ((goal* NEAR/5 state*)) OR TOPIC: ((goal* NEAR/5 specif*)) OR TOPIC: ((goal* NEAR/5 construct*)) OR TOPIC: ((goal* NEAR/5 manag*)) OR TOPIC: ((goal* NEAR/5 direct*)) OR TOPIC: ((goal* NEAR/5 orient*)) OR TOPIC: ((goal* NEAR/5 attain*)) OR TOPIC: ((goal* NEAR/5 achiev*)) OR TOPIC: ((goal* NEAR/5 evaluat*)) OR TOPIC: ((goal* NEAR/5 cent?red)) OR TOPIC: ((goal* NEAR/5 focus*)) Indexes=SCI-EXPANDED,

SSCI, A&HCI, CPCI-S, CPCI-SSH, BKCI-S, BKCI-SSH, ESCI, CCR-EXPANDED, IC Timespan=2000-2021) AND LANGUAGE: (English)

j) SciELO Citation Index

TOPIC: ((Depression or (Anxiety or Anxiety Disorders) or Mood Disorders or (psychological trauma or sexual trauma or stress disorders, post-traumatic or stress disorders, traumatic, acute) or Psychological Distress)) Indexes=SCI-EXPANDED, SSCI, A&HCI, CPCI-S, CPCI-SSH, BKCI-S, KCI-SSH, ESCI, CCR-EXPANDED, IC Timespan=2000-2021) AND TOPIC: ((perspective* or view* or percept* or narrative* or opinion* or barrier* or facilitator* or strength* or weakness* or concern* or understand* or judg* or catalyst* or obstacle* or feedback* or outcome*) or (therapeutic alliance or Decision Making, Shared or (communication or negotiating)) or (Interaction* or Relation* or Partner* or Agreement* or Disagreement* or engag* or disengag* or rapport or collab*)) Indexes=SCI-EXPANDED, SSCI, A&HCI, CPCI-S, CPCI-SSH, BKCI-S, BKCI-SSH, ESCI, CCR-EXPANDED, IC Timespan=2000-2021 AND (TOPIC: ((aim* NEAR/2 behavio*)) OR TOPIC: ((aim* NEAR/2 mental health)) Indexes=SCI-EXPANDED, SSCI, A&HCI, CPCI-S, CPCI-SSH, BKCI-S, BKCI-SSH, ESCI, CCR-EXPANDED, IC Timespan=2000-2021 OR TOPIC: ((target NEAR/2 behavio*)) OR TOPIC: ((target* NEAR/2 mental health)) Indexes=SCI-EXPANDED, SSCI, A&HCI, CPCI-S, CPCI-SSH, BKCI-S, BKCI-SSH, ESCI, CCR-EXPANDED, IC Timespan=2000-2021 OR TOPIC: ((task* NEAR/2 behavio*)) OR TOPIC: ((task* NEAR/2 mental health)) Indexes=SCI-EXPANDED, SSCI, A&HCI, CPCI-S, CPCI-SSH, BKCI-S, BKCI-SSH, ESCI, CCR-EXPANDED, IC Timespan=2000-2021 OR TOPIC: ((personal project NEAR/2 behavio*)) OR TOPIC: ((personal project NEAR/2 mental health)) Indexes=SCI-EXPANDED, SSCI, A&HCI, CPCI-S, CPCI-SSH, BKCI-S, BKCI-SSH, ESCI, CCR-EXPANDED, IC Timespan=2000-2021 OR TOPIC: ((plan NEAR/2 behavio*)) OR TOPIC: ((plan NEAR/2 mental health)) Indexes=SCI-EXPANDED, SSCI, A&HCI, CPCI-S, CPCI-SSH, BKCI-S, BKCI-SSH, ESCI, CCR-EXPANDED, IC Timespan=2000-2021 OR TOPIC: ((striving* NEAR/2 behavio*)) OR TOPIC: ((striving* NEAR/2 mental health)) Indexes=SCI-EXPANDED, SSCI, A&HCI, CPCI-S, CPCI-SSH, BKCI-S, BKCI-SSH, ESCI, CCR-EXPANDED, IC Timespan=2000-2021 OR TOPIC: ((goal* NEAR/5 track*)) OR TOPIC: ((goal* NEAR/5 progess*)) Indexes=SCI-EXPANDED, SSCI, A&HCI, CPCI-S, CPCI-SSH, BKCI-S, BKCI-SSH, ESCI, CCR-EXPANDED, IC Timespan=2000-2021 OR TOPIC: ((goal* NEAR/5 set*)) OR TOPIC: ((goal* NEAR/5 agree*)) OR TOPIC: ((goal* NEAR/5 plan*)) OR TOPIC: ((goal* NEAR/5 consensus)) OR TOPIC: ((goal* NEAR/5 negotiat*)) OR TOPIC: ((goal* NEAR/5 discuss*)) OR TOPIC: ((goal* NEAR/5 propos*)) OR TOPIC: ((goal* NEAR/5 develop*)) OR TOPIC: ((goal* NEAR/5 formulat*)) OR TOPIC: ((goal* NEAR/5 elaborat*)) OR TOPIC: ((goal* NEAR/5 establish*)) OR TOPIC: ((goal* NEAR/5 identif*)) OR TOPIC: ((goal* NEAR/5 write)) OR TOPIC: ((goal* NEAR/5 written)) OR TOPIC: ((goal* NEAR/5 state*)) OR TOPIC: ((goal* NEAR/5 specif*)) OR TOPIC: ((goal* NEAR/5 construct*)) OR TOPIC: ((goal* NEAR/5 manag*)) OR TOPIC: ((goal* NEAR/5 direct*)) OR TOPIC: ((goal* NEAR/5 orient*)) OR TOPIC: ((goal* NEAR/5 attain*)) OR TOPIC: ((goal* NEAR/5 achiev*)) OR TOPIC: ((goal* NEAR/5 evaluat*)) OR TOPIC: ((goal* NEAR/5 cent?red)) OR TOPIC: ((goal* NEAR/5 focus*)) Indexes=SCI-EXPANDED, SSCI, A&HCI, CPCI-S, CPCI-SSH, BKCI-S, BKCI-SSH, ESCI, CCR-EXPANDED, IC Timespan=2000-2021)

Appendix 2: Core Criteria for Quality Assessment of Qualitative Studies

Taken from: Hannes K. Chapter 4: Critical appraisal of qualitative research. In: Noyes J, Booth A, Hannes K, Harden A, Harris J, Lewin S, Lockwood C (editors), *Supplementary Guidance for Inclusion of Qualitative Research in Cochrane Systematic Reviews of Interventions.* Version 1 (updated August 2011). Cochrane Collaboration Qualitative Methods Group, 2011. Available from URL http://cqrmg.cochrane.org/supplemental-handbook-guidance (p.4).

- *"Credibility* evaluates whether or not the representation of data fits the views of the participants studied, whether the findings hold true.
    - Evaluation techniques include having outside auditors or participants validate findings (member checks), peer debriefing, attention to negative cases, independent analysis of data by more than one researcher, verbatim quotes, persistent observation etc.

- *"Transferability* evaluates whether research findings are transferable to other specific settings.
    - Evaluation techniques include providing details of the study participants to enable readers to evaluate for which target groups the study provides valuable information, providing contextual background information, demographics, the provision of thick description about both the sending and the receiving context etc.

- *"Dependability* evaluates whether the process of research is logical, traceable and clearly documented, particularly on the methods chosen and the decisions made by the researchers.
    - Evaluation techniques include peer review, debriefing, audit trails, triangulation in the context of the use of different methodological approaches to look at the topic of research, reflexivity to keep a self-critical account of the research process, calculation of inter-rater agreements etc.

- *"Confirmability* evaluates the extent to which findings are qualitatively confirmable through the analysis being grounded in the data and through examination of the audit trail.
    - Evaluation techniques include assessing the effects of the researcher during all steps of the research process, reflexivity, providing background information on the researcher's background, education, perspective, school of thought etc."
